# A Microscopy Evaluation of Emergence Profile Surfaces of Dental Custom CAD-CAM Implant Abutments and Dental Implant Stock Abutments

**DOI:** 10.3390/jpm14070699

**Published:** 2024-06-28

**Authors:** Daniel Adrian Târtea, Horia Octavian Manolea, Mihaela Ionescu, Oana Gîngu, Marina Olimpia Amărăscu, Adrian Marcel Popescu, Veronica Mercuţ, Sanda Mihaela Popescu

**Affiliations:** 1 Department of Oral Rehabilitation, University of Medicine and Pharmacy of Craiova, 200349 Craiova, Romaniasanda.popescu@umfcv.ro (S.M.P.); 2 Department of Dental Materials, University of Medicine and Pharmacy of Craiova, 200349 Craiova, Romania; 3 Department of Medical Informatics and Biostatistics, University of Medicine and Pharmacy of Craiova, 200349 Craiova, Romania; 4 Department of Engineering and Management of Technological Systems, Faculty of Mechanics, University of Craiova, 200512 Craiova, Romania; oana.gingu@edu.ucv.ro; 5 Department of Dental Morphology, University of Medicine and Pharmacy of Craiova, 200349 Craiova, Romania; marina.amarascu@umfcv.ro; 6 Department of Prosthetic Dentistry, University of Medicine and Pharmacy of Craiova, 200349 Craiova, Romania; ananoki@gmail.com (A.M.P.); veronica.mercut@umfcv.ro (V.M.)

**Keywords:** dental implant custom abutments, CAD/CAM, dental technology, surface analyses

## Abstract

Recently, due to the high demand for dental implants, the use of dental implant stock abutments has increased significantly, especially dental custom CAD/CAM implant abutments milled by dental technicians in their laboratories. The purpose of this study is to analyze the surface quality of the emergence profile of dental custom CAD/CAM implant abutments made by a non-industrial milling machine, compared to original and compatible dental implant stock abutments made by industrial machines. Thirty dental implant abutments were divided into six study lots. Lot 1 (control group): original dental implant stock abutments—industrial machined; lot 2 (study group): compatible dental implant stock abutments—industrial machined; lots 3, 4, 5, and 6 (study groups): compatible custom CAD/CAM dental implant abutments—non-industrial milled with hyperDENT CAM software and Paragon Tools. The Nikon SMZ745T stereomicroscope was used to analyze the emergence profile surface of each dental implant abutment. The structure of the analyzed surfaces did not show significant differences between original and compatible abutments that were industrially machined. As for the customized dental implant abutments, the greatest similarity with the original was obtained for lot 6, and a significant statistical difference was obtained for lot 4. Stepover and Feed Rate parameters of the milling process influenced the surface roughness of the emergence profile for the customized dental implant abutments. The digital technology of non-industrial milling compatible custom CAD/CAM dental implant abutments is reliable and within the correct milling parameters.

## 1. Introduction

The importance of dental implantology as a solution for partial or total edentulism treatment is also due to the CAD-CAM technology. CAD-CAM technology has reached a higher level of development for prosthetic dentistry [1]. Due to the high demand for dental implants, the use of dental implant stock abutments, especially customized ones, has increased significantly. Despite this, the importance of abutment surface treatments has generally been overlooked in patient application. There is insufficient information on the production surface treatment and sterilization techniques of dental implant abutments [2].

One of the important topics approached in implantology is also about the osseointegration of the dental implant, and the success or failure of this aspect depends, on one hand, on the surrounding supporting tissues that anchor the bone implant, providing a protective surface of the soft tissues where the junction with the dental implant abutment is made, and, on the other hand, on the quality of the surface of the dental implant abutment. The dental implant abutment crosses the mucosa and establishes a transmucosal connection between the external environment and the inside of the dental implant [3]. This soft tissue surface surrounding the dental implant abutment, also known as the Emergence Profile, was considered essential to the long-term success of the dental implant [4,5].

Inadequate adhesion of soft tissues to the abutment of a dental implant may result in bacterial infiltration, potentially leading to complications. One way to enhance the attachment of soft tissue cells and reduce biofilm absorption and adhesion is to modify the surface roughness of the emergence profile of the dental implant abutment [6]. This feature allows better mucosal sealing and minimizes peri-implantitis and bone loss. Smooth surfaces reduce the adhesion of macrophages, which are rugophilic and prefer rough surfaces [6]. Several studies have specifically highlighted the effect of surface topography of dental implant abutment materials on bacterial surface interactions [7,8,9].

In their in vivo study, Nascimento et al. made a significant observation. They found that there are both pathogenic and non-pathogenic species colonizing the three surfaces used for the study, namely, dental implant abutment from milled titanium, dental implant abutment from cast titanium, and dental implant abutment from zirconium [7]. This finding is crucial as it sheds light on the diverse microbial colonization on different abutment surfaces. In another study, Teughels et al., analyzing data from 24 papers dealing with the effect of surface roughness on supra/subgingival plaque formation in vivo, concluded that dental implant abutments, dental crowns, and total dentures retain and accumulate plaque [8]. This conclusion emphasizes the importance of maintaining proper oral hygiene to prevent plaque accumulation. In a literature review, Quirynen et al. concluded that the roughness of hard surfaces in the oral cavity significantly impacts the initial adhesion and retention of oral micro-organisms [9]. This review emphasizes the role of surface roughness in microbial adhesion, a key factor in peri-implantitis development.

Clinical research is currently focused on elucidating the mechanisms underlying bacterial adhesion, colonization, and biofilm development on prosthetic abutments and implant surfaces [5,10,11].

The findings of numerous studies have significant implications in the field of prosthodontics and implant dentistry [12,13,14,15,16]. The authors have demonstrated the impact of prosthetic abutment transmucosal design on peri-implant tissue health. In addition to the custom design of the prosthetic abutment, various design types have been used over time, including parallel, concave, convex, convergent, and divergent types. Valente et al. found that the design of the emergence angle can be associated with marginal bone loss, which cannot be directly extrapolated to periimplantitis [12]. Siegenthaler et al. observed in a one-year clinical study that abutments with a concave design stabilized the gingival margin better than convex abutments [13]. A similar study by Souza et al. measured the concave angles of the design, observing that narrow angles are more suitable for minimal bone resorption [14]. Researchers such as Katafuchi et al. and Yi et al. found that the design of the emergence profile and the emergence angle can have a real incidence of peri-implantitis [15,16]. This finding emphasizes the complexity of the relationship between abutment design and peri-implant health and the need for further research. The design of the customized abutments is quite similar to the design of the original abutments, respectively, to those recommended in the literature [17,18]. The clinical results showed that the customized abutments ensured the long-term success of the implants [19].

Various studies have also been conducted on the materials used for personalized prosthetic abutments and their effects on peri-implant tissues [20,21,22,23,24,25,26,27]. Prosthetic abutments are mostly made of titanium and zirconium, but they can also be produced from ceramics and polymers. Linkevicius et al. assessed the stability of peri-implant hard and soft tissues when using abutments made of various materials, such as titanium, gold alloy, zirconium oxide, and aluminum oxide [20]. Their study and others generally did not find significant differences between the materials [21,22,23,24,25,26,27].

Numerous studies have focused on how prosthetic abutments’ surface topography affects interactions with bacteria, which can lead to peri-implantitis [28,29].

Göthberg et al. studied the topography aspect and found no impact on peri-implant inflammation [28]. However, other studies have shown that surface roughness plays a significant role in tissue response and cell adhesion. Wennerberg et al. found that the initial machined surface changes from smooth to rougher after two months of exposure to the oral environment [29].

Brunette et al. [30] conducted one of the first studies on the organized topography of abutment material surfaces. In vitro and in vivo, they demonstrated that repeated micro-grooves could guide cell orientation and spread [30]. Several other authors argue that abutments with micro-grooves promote a uniform and healthy integration of soft and hard tissues [31,32,33,34,35,36,37,38].

One of the manufacturers of dental implants used in our area, Bredent Group, claims to use a concept called Osseo Connect Surface for the implants and components produced. On the processed surface, they claim, there are micro-grooves that support the attachment of soft tissues and prevent plaque formation [39].

To the best of our knowledge, no study in the literature that could be cited analyzes the surface quality of individualized abutments compared to the original Bredent abutments. The purpose of this study is to analyze the surface quality of the emergence profile of custom dental implant abutments milled by a non-industrial milling machine compared to original dental implant stock abutments that were industrial machined. The null hypothesis stated that there are no statistically significant differences between customized dental implant abutments and original dental implant abutments with respect to the surface quality of the emergence profile.

## 2. Materials and Methods

This study evaluated the surface quality of the emergence profile of dental implant abutments of an implant system commonly used in clinical practice in Craiova, Bredent Sky implant system from Bredent Group (Senden, Germany). Six types of dental implant abutments of the same implant system were used. From a prosthetic point of view, the market provides original abutments sold by Bredent but also has parts compatible with original components, including the prosthetic abutment. Some companies sell compatible dental implant stock abutments, and other companies offer a compatible CAD/CAM library of implant connections so that compatible custom dental implant abutments can be milled.

A sample size of 28 abutments was computed using G*Power 3.1.9.7, Heinrich Heine University Düsseldorf, Germany, and considered a significance level α of 0.05 and a power 1-β equal to 0.65—a medium to large effect size value, given that there are very few data available in the literature. Dental implant abutments were divided into six study lots, each one comprising five abutments. The first lot (control group) included the original Bredent Sky stock abutments, the second lot (study group) included the Bredent Sky compatible stock abutments, and the third, fourth, fifth, and sixth lots (study groups) included the Bredent Sky compatible custom CAD/CAM abutments milled in the dental laboratory with different parameters.

**Figure 1:**
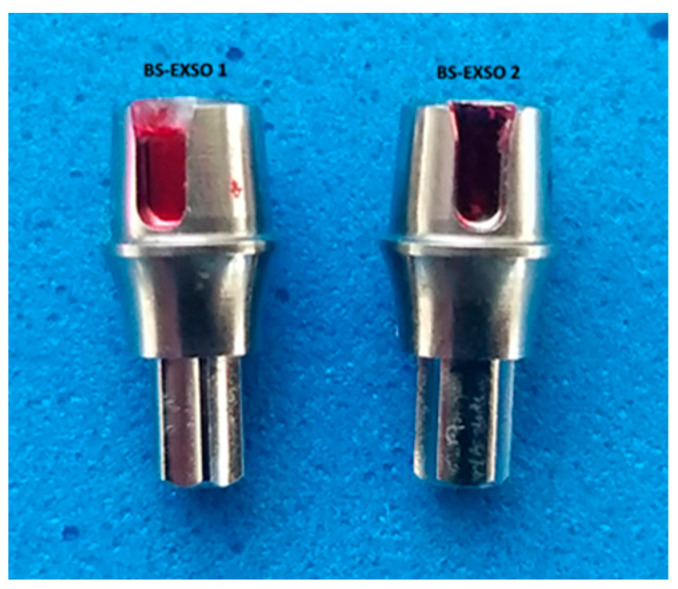
Original dental implant stock abutment.

The control group included the original Bredent Sky Exso (SKYEX003) stock abutments (Figure 1) with a diameter of 4 mm and an emergence profile with a height of 3 mm marketed by Bredent Group (Senden, Germany). In this study, they were named BS-Exso.

**Figure 2:**
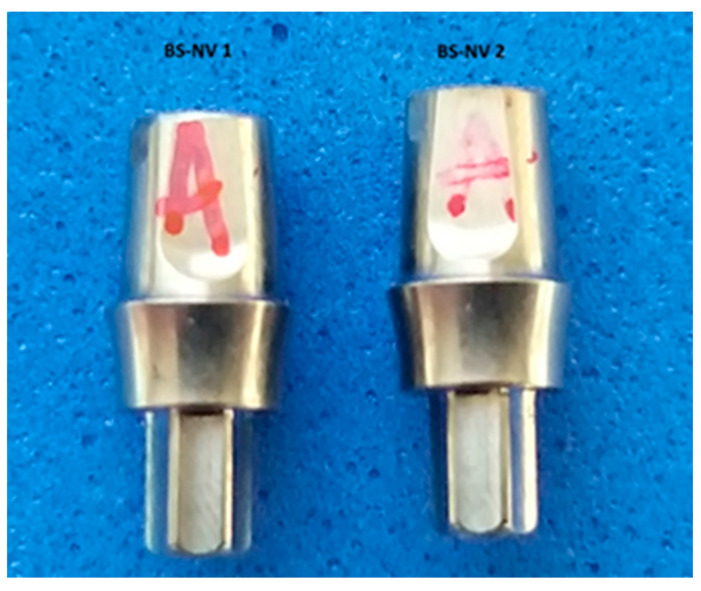
Compatible dental implant stock abutment from NovaMind.

The second lot of dental implant abutments (group 2) was a Bredent Sky compatible stock abutment marketed by NovaMind (Athens, Greece) (Figure 2) with a diameter of 4 mm and an emergence profile with a height of 3.5 mm. In this study, this group was named BS-NM.

**Figure 3:**
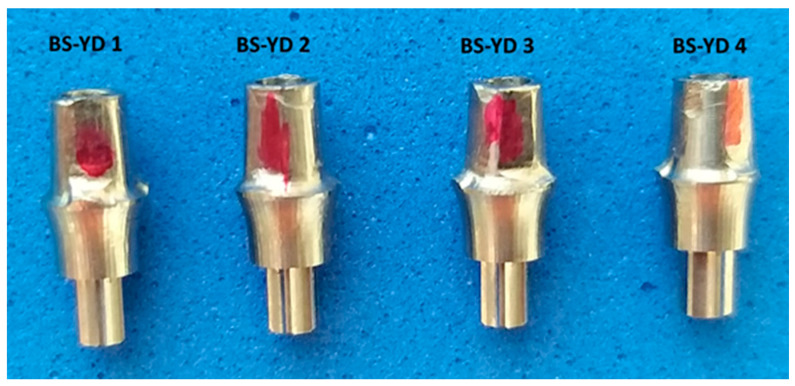
Compatible custom CAD/CAM dental implant abutment.

The next four lots of dental implant abutments (groups 3–6) were milled in the dental laboratory of UGLY DENTURES SRL, Craiova, Romania, with different parameters, using the Bredent Sky compatible CAD/CAM library with a 4 mm platform from the Yenadent company (Istanbul, Turkey) (Figure 3). The 20 dental abutments were randomly assigned to the four study groups using the block randomization technique. In this study, they will be referred to as BS-YD1, BS-YD2, BS-YD3, and BS-YD4. 

For the production of our own Bredent-compatible custom CAD/CAM dental abutment, the following equipment, specialized software, and materials were used:

- Five-axis milling machine with power of 2.7 kW Yenadent D43 (Yena, Istanbul, Turkey);
- hyperDENT Classic v9.4 CAM software (FOLLOW-ME!Technology Group, Munich, Germany;
- Yenadent compatible metal milling tools from Paragon Tools (Paragon Tools, Barcelona, Spain);
- Specialized design software Exocad DentalCAD v3.2 Elefsina (Exocad GmgH, Darmstadt, Germany);
- CAD/CAM Implant Library from Yenadent (Yena, Istanbul, Turkey);
- Titanium disc type 5 grade 23 Nicrallium TA6V ELI diameter of 98.5 mm, and thickness of 14 mm (BCS Dental Alloys, Chassieu, France).

### 2.1. Compatible Custom CAD/CAM Dental Implant Design

**Figure 4:**
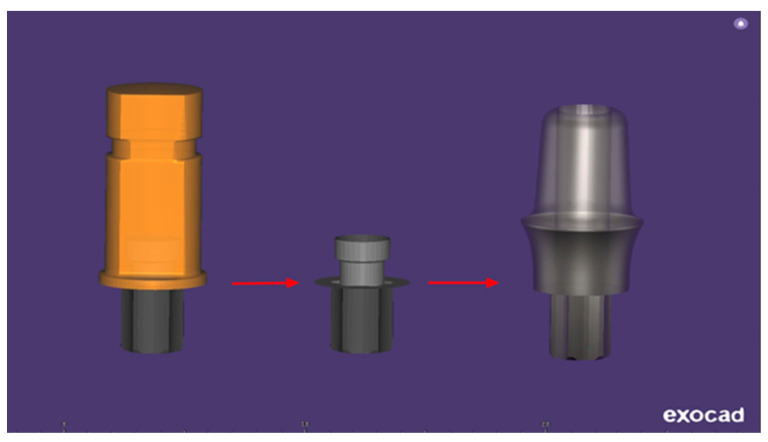
Compatible custom CAD/CAM dental implant abutment designed in Exocad v3.2 Elefsina.

The CAD design of each Bredent Sky-compatible dental implant abutment (Figure 4) was made using the specialized software Exocad DentalCAD 3.2 Elefsina, obtaining a Bredent Sky-compatible dental implant abutment with a platform diameter of 4 mm, a height of the emergence profile of 3.5 mm, and a slightly concave-convergent profile.

### 2.2. Adjustment of Parameters Using the CAM hyperDENT Software and Settings of Tools from Paragon Tools

**Figure 5:**
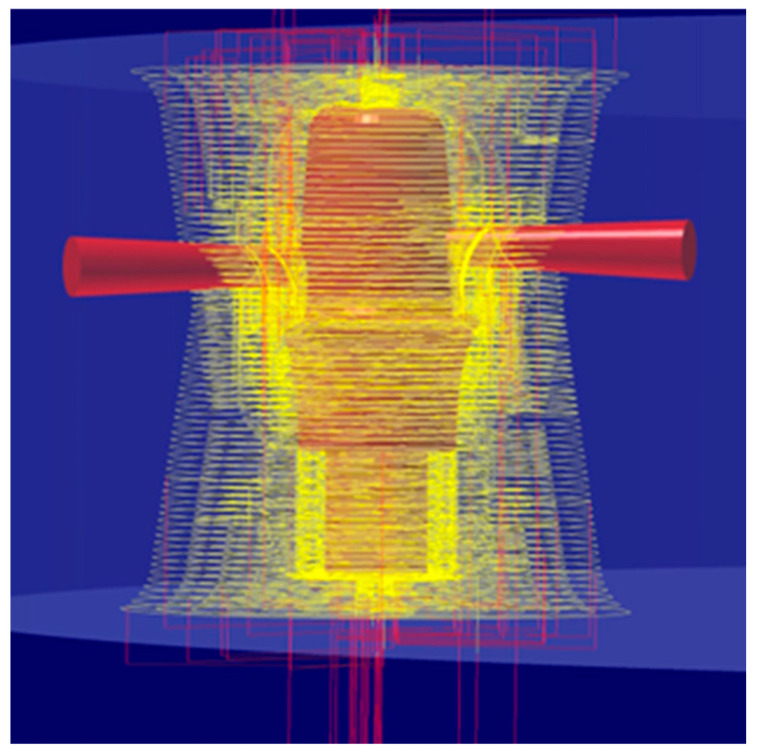
Calculating complex path in hyperDENT.

hyperDENT is currently the leading software in the dental CAM market and offers the possibility for milling cycles from the industrial segment to be efficiently integrated into the dental field, guaranteeing the maximum stability of the production process and top quality. An extremely important thing is also the fact that the parameters of the milling strategies can be adjusted and optimized for each individual machine, regardless of the manufacturing company, offering a reliable calculation with complex and stable paths of the milling tools used for the final product to be a maximum quality [40] (Figure 5).

In order to be able to mill the most common connections of implant systems on the market, the following types of milling tools from Paragon Tools were used: four spherical tools with diameters of 3 mm, 2 mm, 1.5 mm, and 1 mm for the general roughing stages, for finishing from the two occlusal and cavity directions and finishing the emergence profile, two Toric tools, and 2 flat tools for making the screw channel and implant interface.

**Figure 6:**
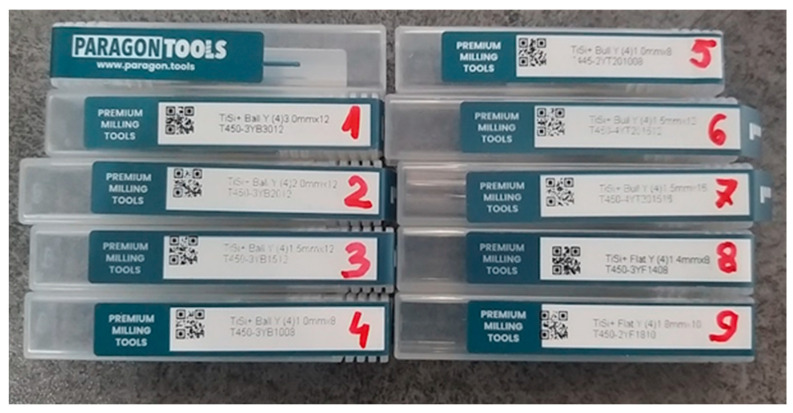
Different types of Paragon tools.

Paragon Tools manufactures high-quality Yenadent compatible milling tools, which is the result of collaboration between experienced cutting tool design engineers, CAD/CAM milling specialists, and milling tools plating experts. These tools have a very hard nanocomposite ceramic coating, the result of a silicon nitride matrix in which titanium and aluminum nitride nanocrystals are deposited, allowing high processing speed for complex titanium alloys, being resistant to wear and oxidation [41] (Figure 6).

Because in hyperDENT, the parameters of the milling strategies may be adjusted, it is possible to compare the surface quality of the emergence profile of the two types of industrially machined dental implant abutments with those milled in the dental laboratory, although there is an obvious difference in processing, industrial machines operating the processing parts by turning, and non-industrial machines operating by milling. The purpose is to check if, by changing the values of the passing steps (Stepover) and the advance speed (Feed Rate) of the spherical tool on the surface of the emergence profile of the dental implant abutment, differences in the quality of the milled surface could be observed. In technical language, it is about the Stepover and Feed Rate [12].

**Figure 7:**
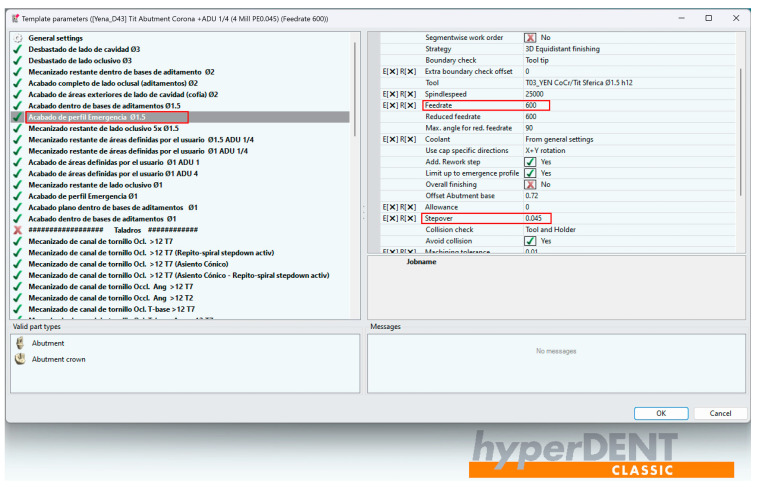
Example of adjusting parameters in hyperDENT.

The BS-YD1-compatible dental implant abutments were milled with the parameters preset by hyperDENT engineers who set the emergence profile to be milled in two stages: the first stage with the spherical milling tool with a diameter of 1.5 mm and Stepover value of 0.1 mm, and the second stage with the spherical milling tool with a diameter of 1 mm and a Stepover value of 0.08 mm, both stages having a Feed Rate of 1200 mm/min (Figure 7, Table 1).

The BS-YD2-compatible dental implant abutments were milled with modified parameters. In the first stage, the spherical milling tool with a diameter of 1.5 mm had a Stepover value of 0.08 mm, and in the second stage, the spherical milling tool with a diameter of 1 mm had a Stepover value of 0.045 mm. Both stages had a Feed Rate of 1200 mm/min.

The BS-YD3-compatible dental implant abutments were milled with modified parameters. In the first stage, the spherical milling tool with a diameter of 1.5 mm had a Stepover value of 0.045 mm, and in the second stage, the spherical milling tool with a diameter of 1 mm had a Stepover value of 0.045 mm. Both stages had a Feed Rate of 600 mm/min.

The BS-YD4-compatible dental implant abutments have been milled to improve the resolution of the implant connection. The fact that Yenadent’s CAD/CAM library offers a Bredent Sky-compatible interface with a low resolution could be identified in the microscope evaluation performed after milling. The milling strategy used was a modified one, so that in the first stage, the spherical milling tool with a diameter of 1.5 mm had a Stepover value of 0.045 mm, and in the second stage, the spherical milling tool with a diameter of 1 mm had a Stepover value of 0.045 mm, both stages having a Feed Rate of 600 mm/min.

### 2.3. Nikon Stereomicroscope SMZ745T

**Figure 8:**
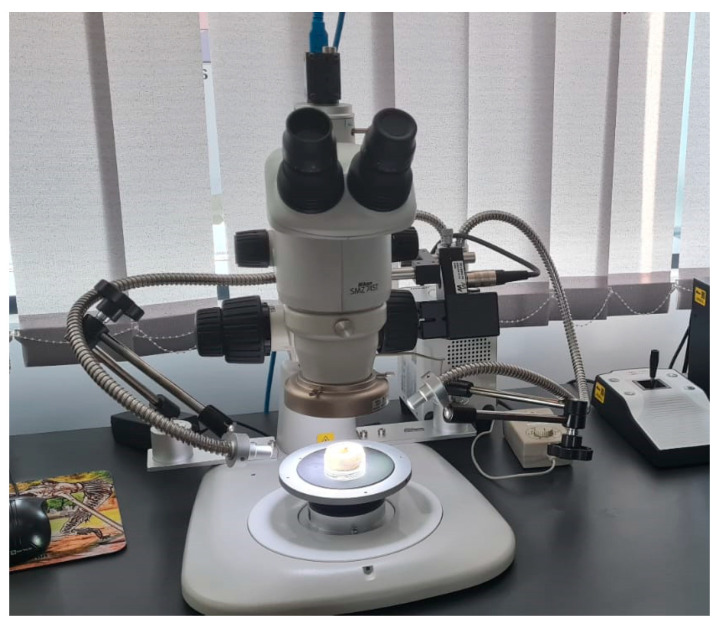
Nikon stereomicroscope SMZ745T.

The surface evaluation of the studied prosthetic abutments was performed by microscopic analysis using the Nikon SMZ745T stereomicroscope (Figure 8), which is recommended for industrial and biomedical applications. It has a magnification of 75× and a working distance of 115 mm, which allows optimal visualization of processing traces on study samples. Real-time EDF (Extended Depth of Field) was used to capture high-resolution images.

**Figure 9:**
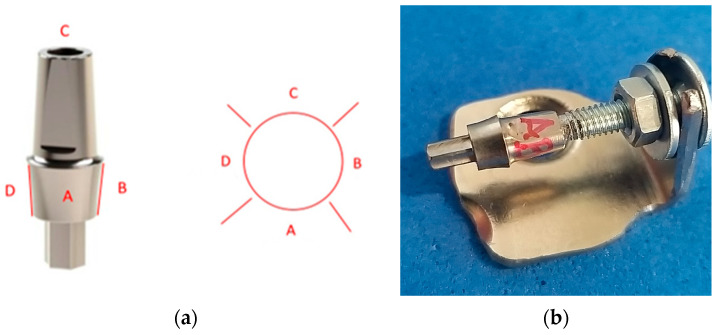
Emergence profile measurements: (**a**) division of areas to be measured; (**b**) device that holds abutments during the measurement process.

For each of the dental implant abutments studied, four faces of the surface of the emergence profile were assigned: Surface A, Surface B, Surface C, and Surface D (Figure 9a).

A device to hold each abutment without touching it was needed to correctly analyze, under the microscope, the four defined surfaces (Figure 9b). The device consisted of several parts: a metal plate shaped like the letter L with a hole on one side for inserting a screw, a screw matching the thickness of the screw channel in the occlusal part of the prosthetic abutment, two washers, and two nuts. Each face was examined under a microscope, and a screwdriver was used to rotate clockwise the abutment fixed to the screw.

After the milling process, custom titanium CAD/CAM abutments need to be cleaned. They are milled wet using an emulsion mixed with water (coolant) to prevent the tools from overheating and ensure better performance. To remove any remaining emulsion and impurities from the milling process, the abutments are ultrasonically cleaned in isopropyl alcohol for 5 min.

**Figure 10:**
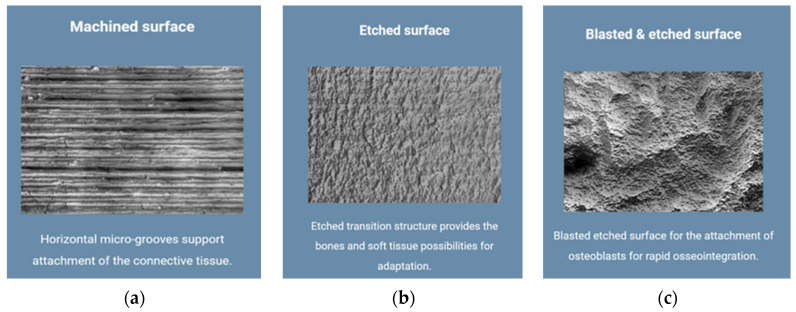
Types of surfaces produced by Bredent Group: (**a**) machined surface; (**b**) etched surface; (**c**) blasted and etched surface.

The evaluation of the surface quality of the emergence profile of the dental implant abutments under study started from the surface quality specified by the manufacturer of the original Bredent Group implant and of the components offered for the prosthetics of the patients through the dental implant. They use a concept called Osseo Connect Surface (OCS)^®^ that contributes to dental implant success by supporting soft tissue attachment and maintaining bone height after implant placement, which is important in promoting optimal osseointegration. On the processed surface of Bredent Sky implants, there are horizontal micro-grooves that support the attachment of soft tissues, creating a protective soft tissue cuff around the implant, thereby preventing plaque formation. In addition to the processed surface with horizontal micro-grooves, the Bredent Group also uses the surface with an etched transition structure to offer bone and soft tissue adaptation possibilities, but also a sandblast-etched surface for attaching osteoblasts for rapid osseointegration (Figure 10a–c) [39].

The evaluation of the surface of the emergence profile of the dental implant abutments was performed at a magnification of 30×, 45×, and 75×, and at the magnification of 75×, several random measurements were made on horizontal micro-grooves observed on the sample surfaces. 

The Ethics Committee of the University of Medicine and Pharmacy of Craiova approved the study by Decision No. 210/10 November 2022.

### 2.4. Statistical Analysis

As previously mentioned, the emergence profile of each abutment was divided into four areas (namely A, B, C and D). Each area was independently analyzed, and the following parameters were determined: the number of micro-cavities, the minimum size (min), and the maximum size (max) of the identified micro-cavities. Thus, three parameters were identified for each face, leading to a total of 12 parameters that characterize an abutment. To perform group comparisons between the six study lots, the following eight parameters were computed for each abutment:

- Total number of micro-cavities, obtained by summing the cavities identified on each face.
- The smallest and the largest cavities, by comparing the min and max values for each face; these values express the exact range of cavities’ sizes present all over the abutment surface.
- Length variation, computed as the ratio between the size range and the maximum length (expressed as percentages); this is a percentual variation of the size of cavities from the entire surface of the abutment.
- The arithmetic mean of the three initial parameters identified for each face; these values express the overall surface of the abutment.
- The variance of the min values and max values measured for the four faces (defined as the degree of spreading of the values within the smallest and largest value).

These eight parameters represent a series of values that provide an overall characterization of each abutment included in the study lot, and they were used for statistical analysis.

Statistical tests were performed using Statistical Package for Social Sciences (SPSS), version 26 (IBM Corp., Armonk, NY, USA) using the parameters computed for each abutment. These continuous variables were expressed as mean ± standard deviation (SD), or medians. Data normality was assessed using the Shapiro–Wilk’s test, and variance was assessed using the Levene’s test of equality of variances. Group comparisons were performed using the Kruskal–Wallis H-test based on the results of the Shapiro–Wilk normality test. Post-hoc analysis was based on Dunn’s (1964) procedure with a Bonferroni correction for multiple comparisons. *p* < 0.05 represented the statistically significance threshold.

## 3. Results

### 3.1. Surface Quality of the Emergence Profile of the Original Dental Implant Abutments (Control Group BS-Exso)

**Figure 11:**
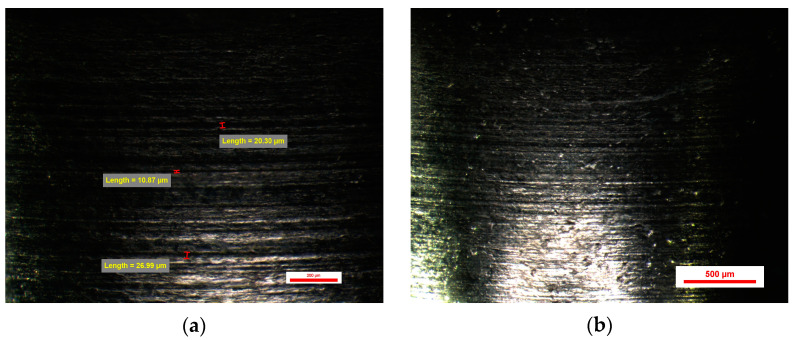

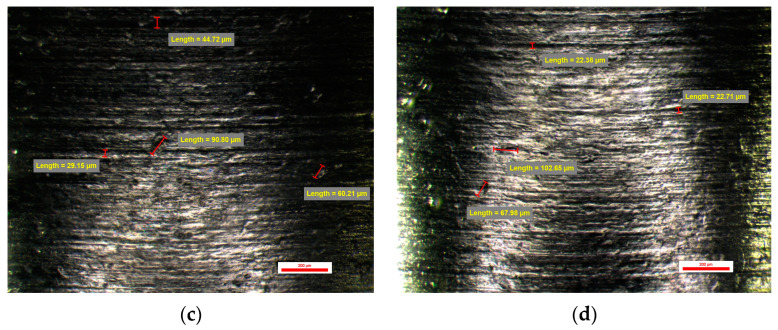
BS-Exso 1 abutment: (**a**) Face A; (**b**) Face B; (**c**) Face C; (**d**) Face D.

The evaluation of the surface of the emergence profile of the original dental implant stock abutments BS-Exso was performed at a magnification of 30×, 45×, and 75×, and at the magnification of 75×, several random measurements were made on horizontal micro-grooves observed on the sample surfaces, each face having a slightly different appearance compared to the other faces of the same dental implant abutment. Micro-grooves show the normal circular appearance of traces of machining tools with different sizes from 10 to 30 µm on all sides of the prosthetic abutments. Certain micro-cavities with dimensions of more than 100 µm have also been observed. All these micro-cavities come from the homogeneity of the titanium material used in the form of a bar in the cutting process rather than from the quality of the working tools (Figure 11).

The abutments from the BS-Exso group had between 51 and 54 microcavities, with a mean value of 13.25 ± 0.68 cavities per face. The minimum value recorded for this study group was 10.87 µm (on face A), and the same abutment had a maximum value of 103.77 µm (on face D). Similarly, the maximum value recorded for this study group was 214.65 µm (on face C), and the same abutment had a minimum value of 20.33 (on the same face C). Min–max percentual variations were comprised between 79% and 91%.

### 3.2. Surface Quality of the Emergence Profile of the Compatible Dental Implant Abutments (Study Group BS-NM)

**Figure 12:**
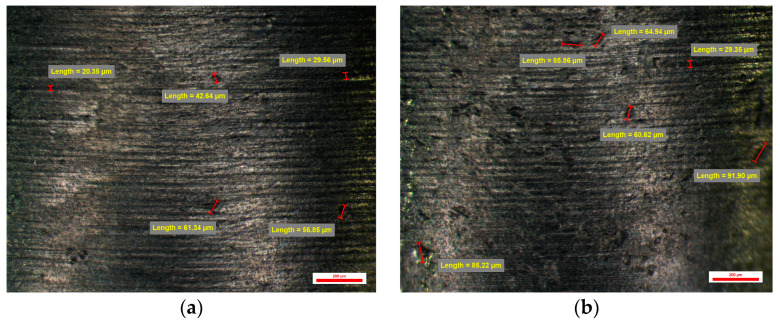

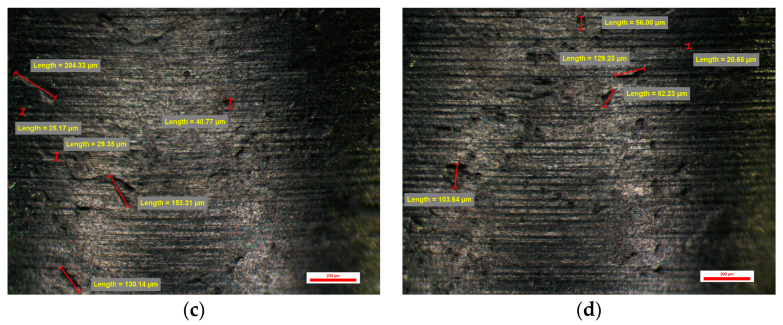
BS-NM 1 abutment: (**a**) Face A; (**b**) Face B; (**c**) Face C; (**d**) Face D.

Also, the evaluation of the surface of the emergence profile of the compatible dental implant stock abutments BS-NM was performed at a magnification of 30×, 45×, and 75×, and at the magnification of 75×, several random measurements were made on micro-grooves observed on the sample surfaces, each face having a slightly different appearance compared to the other faces of the same dental implant abutment as in the case of the original dental implant stock abutments. Micro-grooves have the normal circular appearance of the traces of the machining tools but with a different appearance from the original dental implant abutments due to the type of work tools used, having different dimensions ranging from 22 to 35 microns on all four sides of the compatible dental implant abutments. Also, certain micro-cavities with dimensions of 44, 85, or 100 µm can be observed very rarely and with dimensions over 200 µm (Figure 12).

The abutments from the BS-NM group had a slightly bigger number of cavities, between 55 and 58, with a mean value of 14.00 ± 0.31 cavities per face. The minimum value recorded for this study group was 20.35 µm (on face A), and the same abutment had a maximum value of 204.33 µm (on face C). Similarly, the maximum value recorded for this study group was 243.00 µm (on face C), and the same abutment had a minimum value of 24.72 (on face A). Min–max percentual variations were similar between all abutments from this group, with values comprised between 87% and 90%.

### 3.3. Surface Quality of the Emergence Profile of the Customized Dental Implant Abutments (Study Groups)

The surface evaluation of the emergence profile of the four types of CAD/CAM-compatible prosthetic abutments BS-YD 1, BS-YD 2, BS-YD 3 and BS-YD 4 was performed at 30×, 45× and 75× magnification, and at 75× some random measurements were made on some micro-grooves observed on the surfaces of the samples, each face having a slightly different appearance compared to the other faces of the same type of prosthetic abutment as in the case of the original stock and compatible dental implant stock abutments.

#### 3.3.1. Surface Quality of the Emergence Profile of the Customized Dental Implant Abutments from Lot 3 (Study Group BS-YD1)

**Figure 13:**
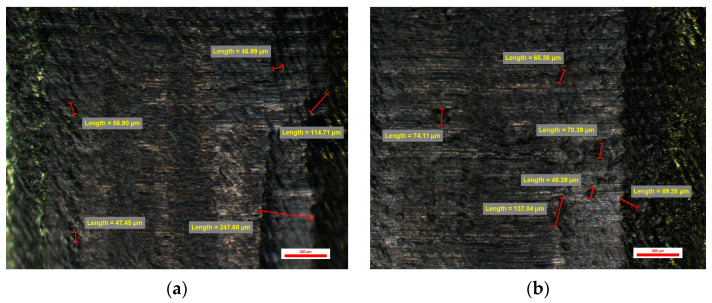

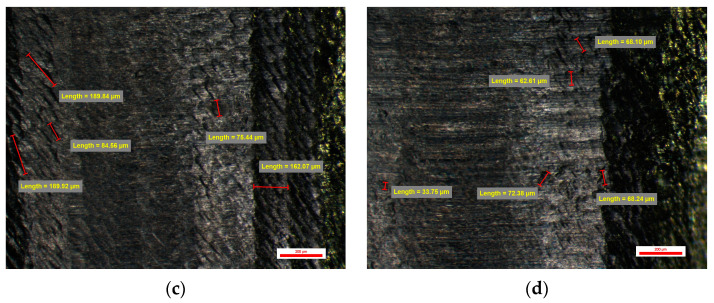
BS-YD 1 abutment: (**a**) Face A; (**b**) Face B; (**c**) Face C; (**d**) Face D.

On the surface of the compatible prosthetic abutments type BS-YD1 milled CAD/CAM with a CAD library whose connection is of low resolution, and the CAM parameters were 0.08 mm Stepover and Feed Rate of 1200 mm/min, vertical and horizontal traces of the passage are observed milling tools specific to vertical milling on the *Z*-axis. These traces leave an appearance of micro-cavities of etched structure with dimensions of 40 µm vertically and over 100 µm horizontally. Micro-cavities with sizes between 30 and 60 µm could also be observed (Figure 13).

One of the abutments from the BS-YD1 group had the smallest number of overall cavities from the entire study lot—namely 38. The maximum was 44, and the mean value was 10.10 ± 0.63 cavities per abutment. The sizes of the cavities were larger than those of the previous groups, with a minimum value of 53.75 µm (on face D), and the same abutment had a maximum value of 189.92 µm (on face C)—which is also the highest maximum value from this group. Min–max percentual variations were similar between all abutments from this group, with values comprised between 34% and 72%.

#### 3.3.2. Surface Quality of the Emergence Profile of the Customized Dental Implant Abutments from Lot 4 (Study Group BS-YD2)

**Figure 14:**
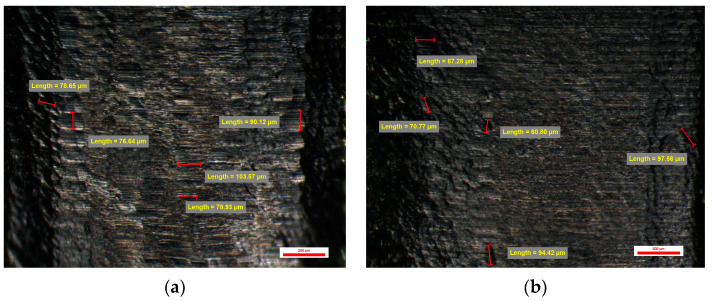

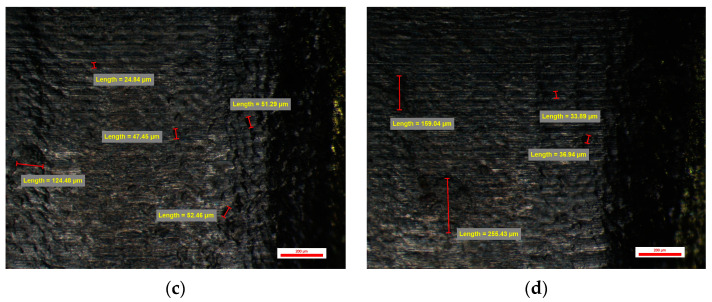
BS-YD 2 abutment: (**a**) Face A; (**b**) Face B; (**c**) Face C; (**d**) Face D.

The same vertical and horizontal traces of the passage of the tools can also be observed on the surface of the compatible dental implant abutments type BS-YD 2. It was also CAD/CAM milled with a CAD library whose connection is of low resolution, and the CAM parameters were 0.045 mm Stepover and a Feed Rate of 1200 mm/min. Changing the CAM parameters shows a slight improvement in the surface appearance of the emergence profile of these compatible dental implant abutments. The micro-cavities observed are between 35 and over 100 µm in size, and the micro-cavities between 25 and 50 µm in size have an etched structure appearance. Micro-cavities with dimensions over 100 µm can also be found (Figure 14).

Abutments from group BS-YD2 had values similar to the other groups, with a mean number of cavities of 10.85 ± 2.07 cavities per face. No extreme values were recorded for this group.

#### 3.3.3. Surface Quality of the Emergence Profile of the Customized Dental Implant Abutments from Lot 5 (Study Group BS-YD3)

**Figure 15:**
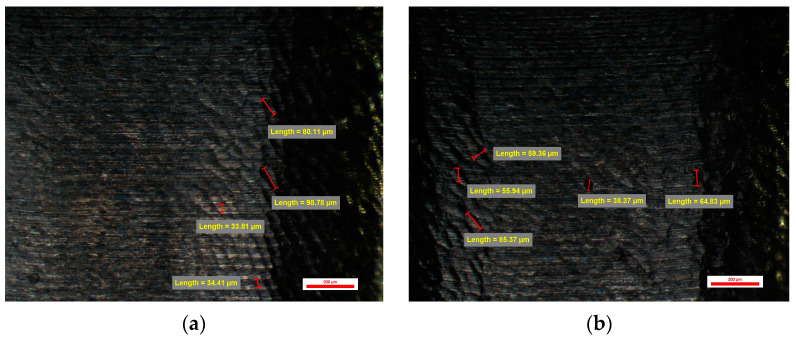

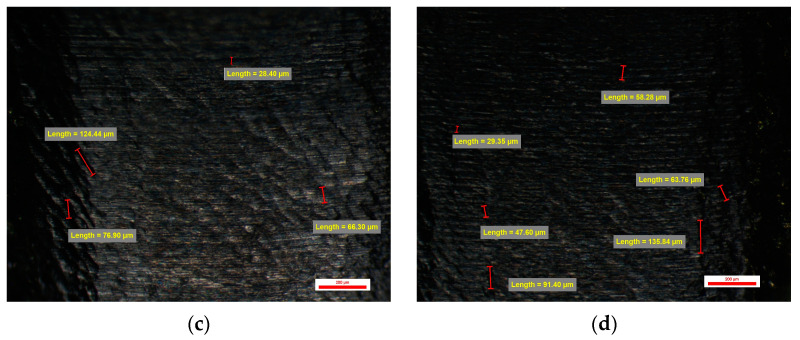
BS-YD 3 abutment: (**a**) Face A; (**b**) Face B; (**c**) Face C; (**d**) Face D.

An even more obvious improvement in the appearance of the emergence profile could be seen in the BS-YD 3 type compatible dental implant abutments. This was made possible by changing the CAM parameters of the Feed Rate, which in this case were 600 mm/min, and the Stepover to 0.045 mm. The CAD quality of the connection was the same as for compatible dental implant abutments. Although vertical and horizontal traces left by the milling tools used can be seen, they are less obvious, leaving a homogeneous appearance of the etched structure surface. Micro-cavities with dimensions between 30 and 90 µm were observed, but also micro-cavities with dimensions between 25 and 45 µm. Micro-cavities with dimensions over 100 µm can also be encountered (Figure 15).

Abutments from groups BS-YD3 and BS-YD4 were quite similar, with a mean number of cavities of 13.45 ± 1.70 cavities per face for group YD3, respectively 13.70 ± 2.18 cavities per face for group YD4. Also, no extreme values were recorded for these groups.

#### 3.3.4. Surface Quality of the Emergence Profile of the Customized Dental Implant Abutments from Lot 6 (Study Group BS-YD4)

**Figure 16:**
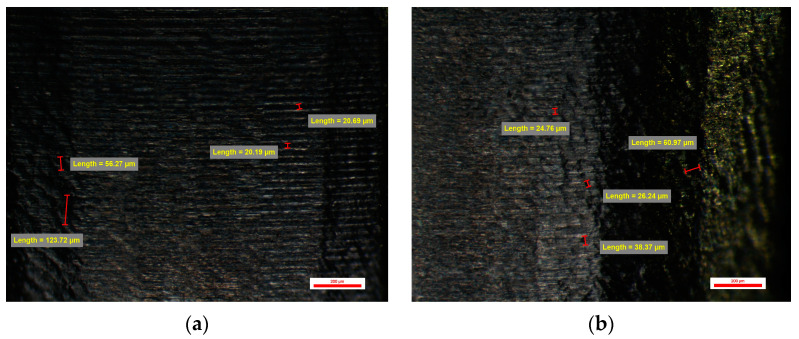

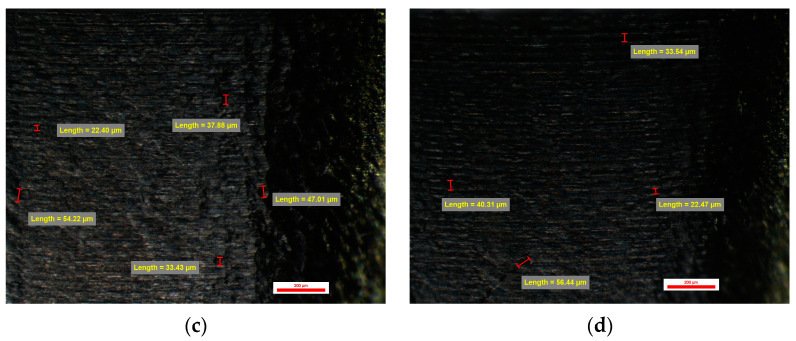
BS-YD 4 abutment: (**a**) Face A; (**b**) Face B; (**c**) Face C; (**d**) Face D.

The BS-YD 4-type CAD/CAM-milled-compatible dental implant abutments with a library whose connection was changed to one whose resolution is high showed the surface quality of the emergence profile as having an obvious qualitative appearance, unlike the other CAD/CAM-milled-compatible dental implant abutments. CAM Feed Rate parameters were 600 mm/min, and Stepover was 0.045 mm. On all surfaces, horizontal micro-grooves with dimensions of 20–22 µm can be observed, as in the case of the original prosthetic abutments, but also micro-cavities specific to spherical burs with dimensions between 33 and 60 µm and other micro-cavities with lengths between 20 and 45 µm that give surface appearance with etched and sandblasted structure. Microcavities with dimensions over 100 µm can also be found (Figure 16).

**Figure 17:**
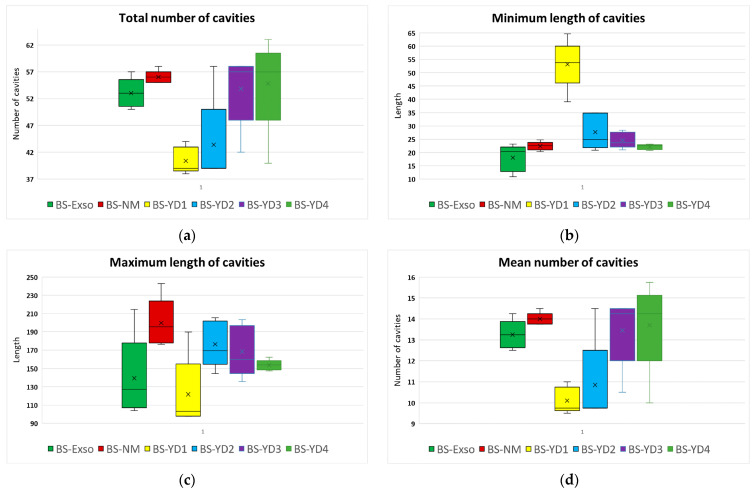

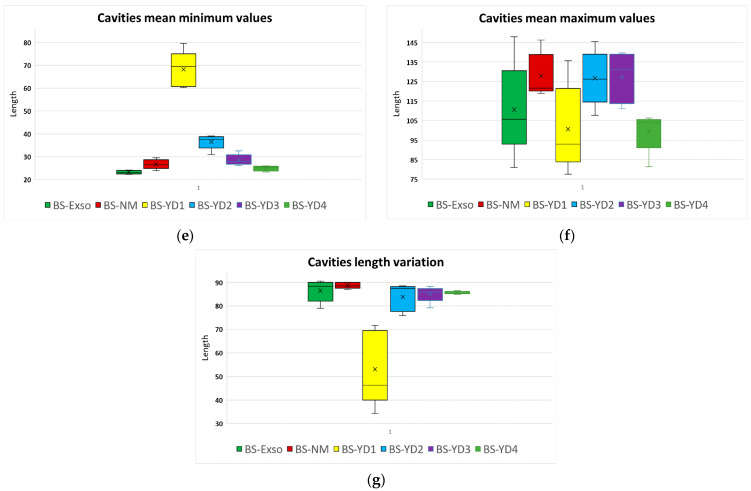
Computed parameters: (**a**) total number of cavities; (**b**) minimum length of cavities; (**c**) maximum length of cavities; (**d**) mean number of cavities; (**e**) mean minimum length; (**f**) mean maximum length; (**g**) length variation.

The random measurement of the dimensions of the observed micro-grooves was done by manual option of the software NIS-Elements D V.5.42, with the mention that these measurements are subjective and approximate, but which helps to make an assessment of the quality of the evaluated surfaces. The measurements within each group were used to compute the parameters that described the overall surface for each abutment. These series were not normally distributed (the results of the Shapiro–Wilk tests provided values > 0.05); the series is presented in Figure 17a–g.

A Kruskal–Wallis test was conducted to determine if there were differences in the above parameters between the six groups of abutment types. Median parameters were statistically significantly different between the different groups for almost all parameters, except max variance, according to Table 2. Subsequently, pairwise comparisons were performed using Dunn’s (1964) procedure. A Bonferroni correction for multiple comparisons was made with statistical significance accepted at the *p* < 0.0033 level. This post hoc analysis revealed statistically significant differences in parameters’ values between different groups, which are mentioned in column “Pairwise comparisons” from Table 2.

The pairwise comparisons from Table 2 and also the charts from Figure 17 indicate that group BS-YD1 (yellow chart) has significantly different values for most of the parameters compared to the original abutments group BS-Exso, leading to significantly different results for almost all parameters for the complete study lot. To further analyze this point, a new Kruskal–Wallis test was conducted after a temporary removal of abutments from group BS-YD1 to determine if there were differences in the above parameters between the five remaining groups of abutments. The results are presented in Table 3.

The results from Table 3 indicate the fact that the presence of group BS-YD1 generated most of the statistically significant differences between groups, as it presented the smallest number of cavities, the smallest percentual length variation, and the highest minimum values. The BS-YD2 group also has significantly larger micro-cavities compared to the original abutments but no other different parameters. Groups BS-YD3 and BS-YD4 do not present statistically significantly different values compared to groups BS-Exso (original abutments) and BS-NM (compatible abutments).

Overall, groups BS-YD2, BS-YD3, and BS-YD4 may be considered similar to the original Bredent abutments and compatible abutments.

## 4. Discussion

The study’s results showed that the surface quality of the emergence profile for customized dental implant abutments milled by a CAD-CAM machine is different from original dental implant abutments and compatible dental implant abutments that were industrially machined, according to the machine’s setting parameters (Stepover and Feed Rate), thus the null hypothesis was rejected. The surface quality of the emergence profile of the customized dental implant abutments milled with a Stepover of 0.045 mm and Feed Rate of 600 mm/min presented great similarity with the surface quality of the original dental implant abutments, although small differences were noted, not statistically significant. It should be noteworthy to know if, in the case of a dental technician trying to mill a customized dental implant abutment, this should have a special design and a surface quality similar to the original abutments [17,18]. Also, there should be clear instructions in any CAM software milling strategy for setting parameters to obtain a specific surface of the emergence profile of the customized dental implant abutment.

Shortly after the implementation of the dental implant rehabilitation solution, there was great interest in studying how titanium dental implant abutments influence the success of implant-prosthetic rehabilitation in long term [42,43].

Numerous studies have highlighted the impact of prosthetic abutment transmucosal design on peri-implant tissue health [5,12]. In the custom design of the prosthetic abutment, dental technicians may use various design types, including concave, convex, parallel, convergent, and divergent, but only a few design types are clinically recommended. Based on four studies in which they analyzed abutments with concave, parallel, convergent, and divergent designs on a total of 230 implants in 145 patients at 12 months, Valente et al. concluded that implants with prosthetic abutments having a concave-convergent profile showed a benefit of 0.209 mm in marginal bone loss [12].

The materials used to make custom prosthetic abutments could affect peri-implant tissue. Various studies have been conducted on different materials to observe what generates inflammation and affects soft tissue [17,22,23].

In a randomized clinical trial conducted by Ferrari et al., forty-seven patients received a total of ninety-seven dental implants. The denture abutments were made of titanium, titanium with titanium nitride coating, and zirconium. After two years of maintenance with a supportive periodontal care program, peri-implant markers were found to be similar for the three materials used, with no significant differences between groups [17,19].

Chokaree et al., in two studies on the properties of materials used to manufacture custom healing abutments, such as PEEK, PMMA, zirconium, composite, and titanium, observed that neither group showed any significant difference in marginal bone modification [22,23].

Regarding the formation of the biofilm, Herrman et al. simulated its growth on different materials at 3 days and 30 days on 14 patients [26]. They created discs with a diameter of 5 mm and a thickness of 2 mm from four different materials: sandblasted and acid-etched titanium simulating the implant surface, machined titanium simulating implant collar, titanium alloy, and zirconium simulating the abutment roughness. The authors concluded that higher bacterial cell counts were found in zirconia abutments compared to machined and titanium alloy [26]. Del Rey et al. have conducted more complex studies on biofilm formation, 10 clinical and 9 in situ, on different materials as alternatives to titanium, such as cobalt-chromium, alumina, polytetrafluoroethylene, gold, and zirconia [27]. In their study, the authors discovered that titanium and zirconia abutments displayed similar levels of bacteria and inflammation, as well as comparable oral health outcomes. However, bacteria levels tended to increase over time. Gold alloys exhibited dense biofilms and greater surface coverage of bacteria than titanium and zirconia. Additionally, all surfaces collected supragingival biofilm [27].

Degidi M. et al., in an in vivo study in which the soft tissue was histologically evaluated after 6 months of implantation around the healing abutments, some untreated (as they came from the manufacturer) and others etched with acid, found that the inflammatory process was greater in the healing abutments acid-etched cures than untreated ones [44]. Also, Fröjd V. et al. evaluated the effect of biofilm formation on the surfaces of three prosthetic abutments, one untreated as it came from the manufacturer, another treated by nanoporous TiO_2_ sauce-gel coating, and another by anodization, and found that there were no major differences in biofilm formation on the three surfaces evaluated [45].

Abrahamsson I. et al., in a study on dogs, consider that a trans-gingival portion with a processed surface can reduce the adhesion of bacteria and the consequent risk of bacterial colonization and inflammation of soft and hard tissues around the implant [46]. In another study, by inserting into the subcutaneous connective tissue of the abdominal wall of rats different titanium surfaces processed by coating with hydrophobic and hydrophilic diamond non-crystalline, they found that the surfaces treated with hydrophilic diamond non-crystalline positively influenced the emptying of the connective tissue [47].

A series of studies have appeared with results regarding soft tissue adhesion to titanium dental implants offering a series of solutions depending on how the dental implant surface is treated, as stated by Gui N. et al. [48] in their study on the effect of ordered and partially ordered topography on bone cell responses, or Rupp F. et al. [49] in their study of dental implant topography characteristics.

Rompen E. et al., in a literature review on the effect of material characteristics, surface topography, and implant components and connections on soft tissue integration, conclude that the results of in vitro and in vivo studies indicate that surface roughness and texture in micrometer range may impact early healing events by influencing epithelial and connective tissue cell attachment, orientation, proliferation, and metabolism, but that studies are limited [50]. In another literature review, Chai et al. found that the issue of how to make the implant surface and its components is not clarified, namely whether there should be a controlled roughness or a smooth surface [51].

A rough surface with micro-grooves of the dental implant abutment can favor epithelial and conjunctival adhesion and thus represent an ideal solution. This tends to favor the formation of a mucous barrier, which is useful to counteract bacterial penetration [31,32,34]. 

Weiner et al. conducted a study comparing how connective tissue, epithelium, and crestal bone behave around micro-grooves and machined implant collars inserted in 6 dogs [31]. The micro-grooved collar was divided into three zones: an upper machined zone of 0.5 mm, a middle zone of 0.7 mm laser micro-grooved with micro-grooves 8 μm wide, and the apical zone of 0.8 mm laser micro-grooved with micro-grooves 12 μm wide. The authors found that, compared to machined surfaces, the micro-grooved surfaces presented minimal soft tissue recession, lower bone loss, and osteoclast activity for all the evaluated periods [31]. Nevins et al. conducted two studies about micro-grooved healing abutments and prosthetic abutments and concluded they could also reattach the soft tissues to their original levels [32,34].

Geurs et al. observed that in a wound produced after replacing a machined abutment with a micro-grooved abutment, the connective tissue attaches to this surface [37].

One of the recent clinical studies conducted by Ahmed et al. [38] showed that micro-groove abutments on which dental restorations are cemented reduced sulcus depth and ridge bone loss compared to machined prosthetic abutments. This led to the conclusion that the use of micro-groove implant collars and micro-groove abutments is a dual strategy that should be used to achieve a more robust and healthy integration of soft and hard tissues [38].

The results of the present study showed that customized dental implant abutments could have an emergence profile surface similar to that of original stock abutments that are industrially manufactured. Dental laboratories have the capability to create custom prosthetic abutments using titanium discs or prefabricated titanium devices. The emergence profile, regardless of the milling method used, is non-industrially milled. Therefore, it’s essential that specialists adjust the milling parameters for each type of milling machine. The emergence profile surface quality depends on the parameters adjusted in CAM Software for the best milling strategy (Stepover and Feed Rate), which are parameters that influence surface roughness. The physical appearance of the machined surface is loaded with concentric circles (feed marks) [52].

Compatible dental implant abutments milled at a non-industrial level are a good alternative to original dental implant abutments machined industrially because, in terms of the surface quality of the emergence profile for the adhesion of soft tissue cells, there are no major differences. The appearance of the surfaces of the original Bredent Sky components, otherwise tested and certified by the Clean Implant Foundation for the independent assessment of the quality of dental implants using accredited laboratories according to DIN EN ISO/IEC 17025, based on a defined protocol [53], presents horizontal micro-grooves after the industrial processing stage which constitute a good support for the attachment of connective tissue [39]. These horizontal micro-grooves are also observed on the emergence profile of compatible dental implant abutments processed industrially, but very importantly, these horizontal micro-grooves are also observed on non-industrial compatible CAD/CAM milled dental implant abutments in the dental laboratory, having even and an appearance of a surface that has been treated, without this having been done.

These studies and observations were mostly performed in vitro; only a few were performed in vivo. Those studies on animals, however, show that evidence of bacterial adhesion to the different types of dental implant abutment surfaces remains unclear, causing a discrepancy between some scientific opinions and daily clinical experience with dental implants. The subject of peri-implantitis has become quite controversial, leading to debate as to whether peri-implantitis should even be considered a disease or whether marginal bone loss would actually be the consequence of the existence of a foreign body placed in the oral cavity [54].

The limitation of this study consisted of the reduced number of abutments included in the study lot.

## 5. Conclusions

The present study aimed to evaluate the surface quality of the emergence profile of non-industrial milled dental implant abutments in a dental laboratory, and comparing them with the emergence profile of the original dental implant stock abutments with compatible ones available on the market.

Thus, it can be concluded that:

- The surface quality of the emergence profile of the non-industrial compatible CAD/CAM milled dental implant abutments represents a safe solution for customized prosthetics on the dental implant; the surface with horizontal micro-grooves observed in the original dental implant stock abutments is also found in the non-industrial milled abutments in the dental laboratory.
- Obtaining a non-industrial-compatible CAD/CAM custom-milled dental implant abutment surface quality is possible in the dental laboratory if it is equipped with CAM software such as hyperDENT that allows the modification of the milling parameters so that the results can be improved to obtain the best results, but also the use of the most suitable milling tools, in our case Paragon Tools.
- The use of specific Stepover (0.045 mm) and Feed Rate (600 mm/min) parameters during the milling process influences the surface roughness of the emergence profile for the customized dental implant abutments, making it possible to obtain a surface similar to the original;
- The use of a higher resolution in milling abutments resulted in a smoother surface of the emergence profile compared to the original abutments.

The CAD library of implant connections provided by dental implant manufacturers, used with the default parameters for abutment design, is not suitable for use in the final milling of custom dental implant abutments (abutments milled with the default parameters were statistically significantly different from the original abutments).

## Figures and Tables

**Table 1:** Milling parameters for study groups 3–6.

Abutments	BS-YD1	BS-YD2	BS-YD3	BS-YD4
Stage 1				
Spherical milling tool diameter (mm)	1.5	1.5	1.5	1.5
Stepover (mm)	0.1	0.08	0.045	0.045
Feed Rate (mm/min)	1200	1200	600	600
Resolution	low	low	low	high
Stage 2				
Spherical milling tool diameter (mm)	1	1	1	1
Stepover (mm)	0.08	0.045	0.045	0.045
Feed Rate (mm/min)	1200	1200	600	600
Resolution	low	low	low	high

**Table 2:** Statistical results regarding the analysis of abutment parameters (expressed in µm), according to the study groups (median values).

Parameter	BS-Exso (1)	BS-NM (2)	BS-YD1 (3)	BS-YD2 (4)	BS-YD3 (5)	BS-YD4 (6)	*p* *	Pairwise Comparisons **
Total no of cavities	53.00	56.00	39.00	39.00	57.00	57.00	0.017	3–6
Min size	20.33	22.51	53.75	24.87	23.62	22.56	0.003	1–3, 3–6
Max size	127.33	195.50	103.27	169.43	159.74	153.72	0.022	2–3
Length variation	88%	88%	46%	87%	86%	85%	0.004	1–3, 2–3
Mean no of cavities	13.25	14.00	9.75	9.75	14.25	14.25	0.017	-
Mean Min size	23.03	26.46	69.56	37.55	27.92	25.16	<0.0005	1–3, 1–4, 3–6
Mean Max size	105.75	121.71	92.89	126.26	131.03	104.07	0.020	-
Min variance	9.87	20.57	159.99	46.76	15.72	8.30	0.021	1–3, 3–6
Max variance	270.93	1679.51	199.13	628.19	936.72	951.90	0.071	-

* Kruskal–Wallis H-test. ** Statistically significant pairwise comparisons (group numbers).

**Table 3:** Statistical results regarding the analysis of abutment parameters (expressed in µm), according to the modified study groups—after removal of the BS-YD1 group (median values).

Parameter	BS-Exso (1)	BS-NM (2)	BS-YD2 (4)	BS-YD3 (5)	BS-YD4 (6)	*p* *	Pairwise Comparisons **
Total no of cavities	53.00	56.00	39.00	57.00	57.00	0.146	-
Min size	20.33	22.51	24.87	23.62	22.56	0.066	-
Max size	127.33	195.50	169.43	159.74	153.72	0.057	-
Length variation	88%	88%	87%	86%	85%	0.139	-
Mean no of cavities	13.25	14.00	9.75	14.25	14.25	0.146	-
Mean Min size	23.03	26.46	37.55	27.92	25.16	0.001	1–4, 4–6
Mean Max size	105.75	121.71	126.26	131.03	104.07	0.020	-
Min variance	9.87	20.57	46.76	15.72	8.30	0.518	-
Max variance	270.93	1679.51	628.19	936.72	951.90	0.114	-

* Kruskal–Wallis H-test. ** Statistically significant pairwise comparisons (group numbers).

## Data Availability

The authors declare that the data of this research is available from the corresponding authors upon reasonable request.
